# Identify schizophrenia using resting-state functional connectivity: an exploratory research and analysis

**DOI:** 10.1186/1475-925X-11-50

**Published:** 2012-08-16

**Authors:** Yan Tang, Lifeng Wang, Fang Cao, Liwen Tan

**Affiliations:** 1 Biomedical Engineering Laboratory, School of Geosciences and Info-Physics, Central South University, Changsha, Hunan, 410083, Peoples Republic of China; 2 2 Mental Health Institute, the Second Xiangya Hospital of Central South University, Changsha, Hunan, 410011, Peoples Republic of China

**Keywords:** Schizophrenia, fcMRI, Resting-state, Multivariate pattern analysis, Reconstruction

## Abstract

**Background:**

Schizophrenia is a severe mental illness associated with the symptoms such as hallucination and delusion. The objective of this study was to investigate the abnormal resting-state functional connectivity patterns of schizophrenic patients which could identify furthest patients from healthy controls.

**Methods:**

The whole-brain resting-state fMRI was performed on patients diagnosed with schizophrenia (n = 22) and on age- and gender-matched, healthy control subjects (n = 22). To differentiate schizophrenic individuals from healthy controls, the multivariate classification analysis was employed. The weighted brain regions were got by reconstruction arithmetic to extract highly discriminative functional connectivity information.

**Results:**

The results showed that 93.2% (*p* < 0.001) of the subjects were correctly classified via the leave-one-out cross-validation method. And most of the altered functional connections identified located within the visual cortical-, default-mode-, and sensorimotor network. Furthermore, in reconstruction arithmetic, the fusiform gyrus exhibited the greatest amount of weight.

**Conclusions:**

This study demonstrates that schizophrenic patients may be successfully differentiated from healthy subjects by using whole-brain resting-state fMRI, and the fusiform gyrus may play an important functional role in the physiological symptoms manifested by schizophrenic patients. The brain region of great weight may be the problematic region of information exchange in schizophrenia. Thus, our result may provide insights into the identification of potentially effective biomarkers for the clinical diagnosis of schizophrenia.

## Background

Schizophrenia is the most chronic and disabling of the severe mental disorders [1]. Until now, there is no definitive standard in the diagnosis of schizophrenia, which is mainly based on patient interviews and symptom history [2].

It has been reported that patients diagnosed with schizophrenia have showed the functional disconnections distributed in whole brain areas [3,4], suggesting that schizophrenia may arise from abnormalities in a distributed network of brain regions. Using the seed-based region-of-interest correlation analysis, Woodward et al. [5] discovered that RSNs were differentially affected in schizophrenic patients, and Sridharan et al. [6] demonstrated that the anterior insula played a causal role in functions switching between the central-executive network(CEN) and default mode network(DMN). Nevertheless, the seed-based method, which only focuses on a handful of brain regions of interest and doesn’t examine functional connectivity patterns on a whole-brain scale, may not be a particularly effective approach to reveal the pathological mechanism that leads to schizophrenia. Moreover, several studies have suggested that rest-based functional analyses can not only detect more complete and accurate information of functional connectivity [7,8], but also be easier to perform resting-state neuroimaging in schizophrenic patients in contrast to task-related imaging. Therefore, we used whole-brain, resting-state fMRI data for the analyses performed in this study.

Multivariate classification algorithms, which can analyze the whole-brain fMRI data, have been increasingly used to investigate physiological disturbances that lead to mental illnesses [2,9] by extracting additional information from high-dimensional neuroimaging data and identifying potential neuroimaging-based biomarkers to differentiate patients from healthy controls. To improve the performance of the classification algorithms, an increasing number of researchers are now using nonlinear dimensionality reduction methods such as local linear embedding (LLE) [10,11], and nonlinear classifiers such as c-means [12] and neural networks [1], to differentiate patients from healthy controls. However, these reports failed to reconstruct and identify distinct features that quantitatively and qualitatively contributed to the classification of disease.

Here, we used a data-driven method for disease classification that integrated principle component analysis (PCA) and support vector machine (SVM) to extract spatiotemporal patterns associated with schizophrenia from resting-state functional connectivity. Most importantly, we reconstructed brain regions that exhibited functional abnormalities, which might lead to schizophrenia.

## Materials and methods

### Participants

In this study, all subjects were right-handed, native Chinese speakers. Twenty-two schizophrenic patients were recruited from the Department of Psychiatry, Second Xiangya Hospital of Central South University in Changsha, China. Patients were evaluated based on a patient version of the Structured Clinical Interview for DSM-IV (Diagnostic and Statistical Manual of Mental Disorders, Fourth Edition) and satisfied the DSM-IV diagnostic criteria for schizophrenia. Patients were not included if they had a prior history of major head trauma, alcohol or drug abuse. Among the schizophrenic patients, 5 were drug naive, while the remainder were receiving antipsychotic medications at the time of image acquisition (risperidone [n = 8, 2–4 mg/day], clozapine [n = 3, 200–350 mg/day], quetiapine [n = 4, 400–600 mg/day], aripiprazole [n = 1, 25 mg/day], and sulpiride [ n = 500 mg/ day ]).

Healthy volunteers were recruited via advertisement and were eligible for participation if they did not have a prior history of mental illness and a family history of any psychiatric disorders. The exclusion criteria for controls were similar to those for schizophrenic patients. Schizophrenic patients and healthy control subjects were closely matched regarding age, gender and education (Table 1). All participants completed the neuropsychological tests of vigilance and working memory on the days before scanning. In addition, the symptoms of all the patients were assessed with the Positive and Negative Symptoms Scale (PANSS) [13]. Test results are also presented in Table 1.

**Table 1:** Characteristics of the participants in the study

**Variable**	**Patients (n = 22)**	**Healthy controls (n = 22)**	**t/*χ*2 value**	**p-value**
Age (years)	24.54 ± 6.70	26.09 ± 6.47	-0.78	0.44
Sex(M/F)	15/7	15/7	0.00	1.00
Education(years)	12.14 ± 3.37	12.73 ± 2.66	-0.65	0.52
Duration(years)	3.21 ± 3.34			
PANSS	75.64 ± 12.91			
Logical Memory Immediate	8.86 ± 4.85	14.32 ± 3.43	-4.31	0.00
Logical Memory Delay	6.95 ± 4.73	13.36 ± 3.61	-5.06	0.00
Saving Score	0.70 ± 0.27	0.94 ± 0.15	-3.57	0.001

Both the patients and their guardians agreed to participate in the study and written informed consents were obtained from all subjects after a detailed explanation of the study. This study was approved by the Ethics Committee of the Second Xiangya Hospital, Central South University.

### Procedure

During the experiments, subjects were explicitly instructed to stay relaxed, keep their eyes closed, remain awake and move as little as possible. After each scan, we confirmed that subjects were awake during the experiments.

### Imaging data acquisition

Imaging was performed at the Second Xiangya Hospital of Central South University on a 1.5 T GE Signa System (GE Signa, Milwaukee, Wisconsin, USA). Foam pads with a standard birdcage head coil were used to fix the subject’s head. fMRI data were collected using a gradient-echo EPI sequence. The imaging parameters were as follows: TR =2000 ms, TE =40 ms, FOV =24 × 24 cm^2^, Flip Angle = 90°, and matrix = 64 × 64. Whole-brain volumes were acquired with 20 contiguous 5-mm-thick transverse slices with a 1-mm gap. Each resting-state functional imaging scan lasted for 6 minutes. T1-weighted images (TR = 2000 ms, TE = 7.5 ms, TI = 750 ms) were taken at the same location as the functional images and were acquired prior to functional scanning to obtain the subject’s anatomical information.

### Data analysis

#### Preprocessing

All neuroimaging data were preprocessed by using the statistical parametric mapping software package (spm8, Wellcome Department of Cognitive Neurology, Institute of Neurology, London, UK, http://www.fil.ion.ucl.ac.uk/spm) and resting-state fMRI Data Analyze Toolkit (REST version 1.5; http://http://www.restfmri.net). The calculations were executed in Matlab 7.1 (MathWorks, Sherborn, MA). The first 5 volumes of each functional series were discarded because of magnetic saturation effects, and the remaining images were preprocessed by using the following steps: re-alignment for head motion, spatial normalization to the standard MNI space and spatial smoothing with an 8-mm full-width at half maximum (FWHM) Gaussian kernel. In this study, all subjects had less than 1 mm translation in the x-, y- or z-axis and less than 1° of rotation in each axis. Then we used the REST toolkit to remove the linear trend of time courses and performed temporal band-pass filtering (0.01 Hz < f < 0.08 Hz).

#### Analysis

According to the anatomically labeled template from the mask of WFU_PickAtlas (Department of Radiologic Sciences, WFU School of Medicine, Medical Center Blvd. Winston-Salem, NC, US http://www.fmri.wfubmc.edu/cms/software), the registered fMRI volumes with the MNI template divided the cerebrum into 90 regions (45 regions within each hemisphere)[14]. Regional mean time series were obtained for each subject by averaging the fMRI time series over all the voxels in each of the 90 regions. Before the correlation analysis, the nuisance covariates, including head motion parameters, global mean signals, white matter signals and cerebrospinal fluid signals, were regressed from the images. Although this technique has been suggested to result in a negative mean correlation value during functional connectivity analysis [15], regression analyses may enhance the detection of system-specific correlations and improve the correspondence between resting-state correlations and anatomy [16]. Next, we used Pearson’s correlation coefficients to evaluate the functional connectivity between each pair of regions. Thereby, we obtained a resting-state functional network that was expressed as a 90x90 symmetrical matrix for each subject. Then, by removing the 90 diagonal elements, we determined the upper triangular elements of the functional connectivity matrix to be the classification features. Thus, we obtained 4005 dimensional feature vectors as the feature space for classification [12].

#### Classifications with highly discriminative features

**Figure 1:**
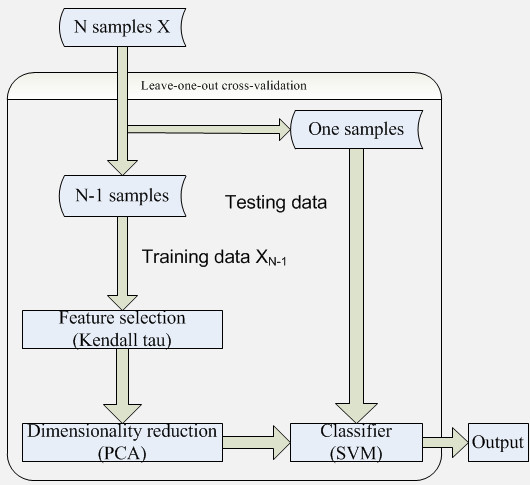
Flow chart of the algorithm.

We completed feature extraction of the whole brain’s functional connectivity pattern according to previously described methods for data processing. Followed, Fisher’s z-transform was applied to the correlation values to ensure normality. Here, we discussed the main procedures of classification between schizophrenic patients and healthy controls, consisted of four parts: feature selection, dimensionality reduction, classification and reconstruction (Figure 1).

After we obtained 4005 feature vectors , considering the discrete nature of these feature vectors, we used the Kendall tau rank correlation coefficient *τ*[17] to extract features, which displayed highly discriminative power, and then obtained the feature space.

Suppose there are *m* samples in the patient group and *n*samples in the control group. Let *x*_*i*,*j*_ denotes the functional connectivity feature *i* of the *j*_*th*_ samples, and *y*_*j*_ denotes the class label of this sample (-1 for patients and +1 for controls). The Kendall tau correlation coefficient of the functional connectivity feature *i* can be defined as

(1) $$\tau_{i}=\frac{n_{c}-n_{d}}{m\times n}$$

Where *n*_*c*_ and *n*_*d*_ are the number of concordant and discordant pairs respectively. Because we don’t consider the relationship of two samples that belong to the same group, the total number of sample pairs is *m* × *n*. For a pair of observation dataset {*x*_*i*,*j*_, *y*_*j*_} and{*x*_*i*,*k*_, *y*_*k*_}, it’s a concordant when

(2) $$\mathrm{sgn}\left({\text{x}}_{i,j}-{\text{x}}_{i,k}\right)=\mathrm{sgn}\left(y_{j}-y_{k}\right)$$

Correspondingly, it’s a disconcordant pair when

(3) $$\mathrm{sgn}\left({\text{x}}_{i,j}-{\text{x}}_{i,k}\right)=-\mathrm{sgn}\left(y_{j}-y_{k}\right)$$

Thus, the positive correlation coefficient, *τ*_*i*_, represents the *i*_*th*_ functional connectivity that exhibits a significant decrease in the patient group compared with the control group, while the negative correlation coefficient *τ*_*i*_, represents the *i*_*th*_ functional connectivity that exhibits a significant increase in the patient group compared with the control group. Moreover, this difference increases substantially when the absolute value of the Kendall correlation coefficient *τ* is larger. Above all, the Kendall tau rank correlation coefficient *τ* provides a distribution-free test of independence between two variables that measures the relevance of each feature of classification. Therefore, we used *τ* as a foundation for extracting feature vectors. To get optimal feature number of the final feature space for classification, the selection was performed only on training data in leave-one-out cross-validation (LOOCV) [18].

Then, we used PCA to separately reduce the dimensionality and obtained a subspace, ${\tilde{\text{X}}}^{\text{T}}=({\tilde{\text{x}}}_{1}$…$,{\tilde{\text{x}}}_{d})$, where d is the number of the reduced dimensionality. This mathematical description is provided in the Additional file 1. Here,

(4) $$\tilde{\text{X}}={\text{U}}^{\text{T}}\left(\text{X}-\bar{\text{X}}\right)$$

Where $\bar{\text{X}}$ was the mean vector of *X*^*T*^ = (*x*_1_…, *x*_*d*_). Usually, *U* was made up of *d* eigenvectors with the first *d* largest eigenvalues of the covariance matrix.

Finally, we adopted linear SVM to classify the feature. Its mathematical description is also given in the Additional file 2. Here,

(5) $$\text{Y}={\text{w}}^{\text{T}}\tilde{\text{X}}-\text{b}$$

Where Y is the result of SVM. When Y > 0, we could classify *x*_*i*_ as a positive sample and otherwise a negative sample.

Because of the limited number of samples in our study, we used LOOCV strategy [19] to estimate the generalized performance of our classifier. Thus, for N samples, the LOOCV would train the classifier N times. The performance of the classifier could be quantified by using Generalization Rate GR, Sensitivity SS and Specificity SC based on the results of LOOCV,

(6) $$GR=\frac{\text{T}P+\text{T}N}{\text{T}P+FN+\text{T}N+FP}$$

(7) $$SS=\frac{\text{T}P}{\text{T}P+FN}$$

(8) $$SC=\frac{\text{T}N}{\text{T}N+FP}$$

where TP, TN, FP and FN, denote the number of correctly predicted schizophrenic patients, correctly predicted control subjects, control subjects incorrectly classified as schizophrenic patients and schizophrenic patients incorrectly classified as control subjects, respectively. Note that Generalization Rate GR, Sensitivity SS and Specificity SC represent the overall proportion of samples that are correctly predicted, the proportion of patients correctly predicted and the proportion of controls correctly predicted, respectively.

#### Reconstruction

After classification, we reconstructed features and brain regions for functional connectivity. The process of reconstruction is an inverse process of classification. In fact, we combined the Equation (4) and Equation (5) in the classifier training procedure, and then we obtained the following Equation:

(9) $${\text{Y}}_{\text{i}}={\text{w}}^{\text{T}}\left({\text{U}}^{\text{T}}\left({\text{x}}_{i}-{\bar{\text{x}}}_{i}\right)\right)-\text{b}={\left(\text{Uw}\right)}^{\text{T}}\left({\text{x}}_{i}-{\bar{\text{x}}}_{i}\right)-b$$

Suppose* Λ* = *Uw* = (*λ*_1_, …, *λ*_*d*_)^*T*^, then the absolute value of the element *λ*_*j*_(*j* ∈ 1, …, *D*) represents the contribution of the corresponding feature to the discriminative score. Hence, these values can be used to measure the discriminative power of the corresponding feature, called feature weight. Because of the LOOCV, we initially computed the weight, *Λ*_*i*_ in every LOOCV.

After LOOCV, we averaged the absolute value of *Λ*:

(10) $$\Lambda =(\left|{\text{Λ}}_{1}\right|+\left|{\text{Λ}}_{2}\right|+\ldots +\left|{\text{Λ}}_{N}\right|)/N$$

where *N* is the number of subjects. The final step for reconstruction was to compute the weight of each brain region. For instance, the weight of brain region A was computed as follows:

Step 1: Determine all the brain regions that have functional connections with *A* and obtain their corresponding feature weights.

Step 2: Assign half of each feature weight to *A*.

Step 3: Sum all of the corresponding half-weights for each feature. This sum is the weight of brain region *A*.

#### Permutation tests

Many studies assess the performance of a classifier by conducting permutation tests [20-23]. In this study, we used this approach to assess the statistical significance of all the LOOCV results. For each LOOCV, the classification labels of the training data were randomly permuted 1000 times. Afterwards, the entire classification operation, including the feature selection, dimensionality reduction, and SVM, was carried out on every set of randomized class labels. The GR is defined as the classification prediction rate obtained by the classifier trained on the randomly re-labeled classification labels, and the GR0 is the classification prediction rate obtained by the classifier trained on the real class labels. The *p*-values reported for accuracy represent the probability of GR being no less than GR0. When *p* < 0.05, it was assumed that the classifier could reliably assign labels from the data.

## Results

### Classification results

**Figure 2:**
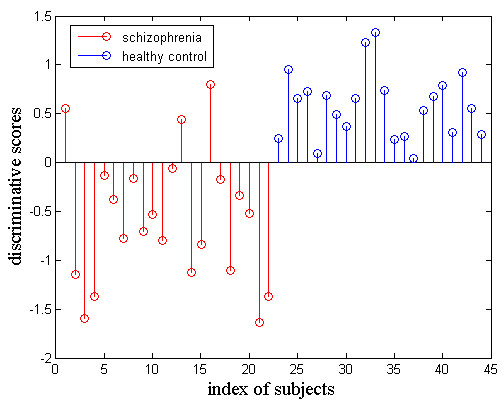
**The discriminative scores of all subjects.** The first 22 samples represented schizophrenic patients. The remaining samples represented healthy controls.

Using 550 features in the feature selection, SVM clustering results demonstrated that the final correct classification rate (generalization rate, GR) was 93.2% (100% of healthy controls and 86.4% of schizophrenic patients) (Figure 2).

**Figure 3:**
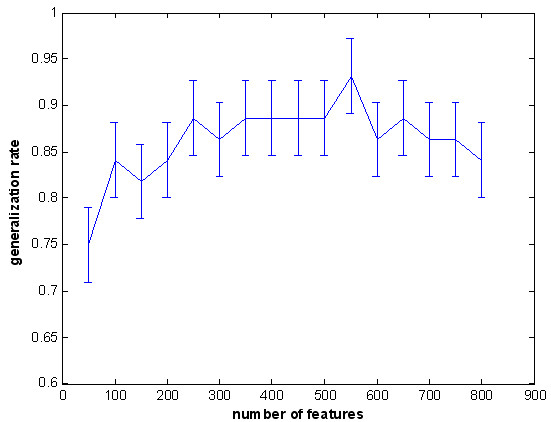
**The curve of the generalization rate to the number of features.** The horizontal axis represents the number of selected features and the vertical axis represents the generalization rate.

To estimate the effect of the number of selected features on the performance of the classifier, we repeated this calculation with a varying number of different features in the feature selection [12,20]. We found that the classifier’s best performance was achieved at 550 (Figure 3) by examining a range of feature numbers (from 50 to 800). Therefore, we selected 550 as the optimal feature number of the final feature space for classification in LOOCV.

The similar methods of the selection for optimal feature number of the final feature space for classification also were used to choose the dimension for PCA and C for SVM. We repeated this calculation with a range of different values (dimension: 2-20 and C: 0.005:0.05:2). Then, we chose the value when the classifier achieved its best performance (that is generalization rate).Therefore, we selected 6 as the optimal dimension for PCA and 0.255 as C for SVM. Several studies selected the parameters in this way, just as Besga et al. [18] and Dosenbach et al. [20] selected the number of features in LOOCV.

Altered resting-state functional connections in schizophrenia.

Because the performance of the classifier was tested with an LOOCV strategy, a given functional connectivity feature might display a different discriminative power in different iterations of the LOOCV. Based on statistical analyses, we found that more than 68% of the functional connectivity features had positive Kendall tau correlation coefficients, indicating that more than 68% of functional connectivities decreased in schizophrenic patients compared with controls. Moreover, the majority of the functional connectivity, which was characterized by a high discriminative power, decreased in schizophrenic patients compared with healthy controls.

**Figure 4:**
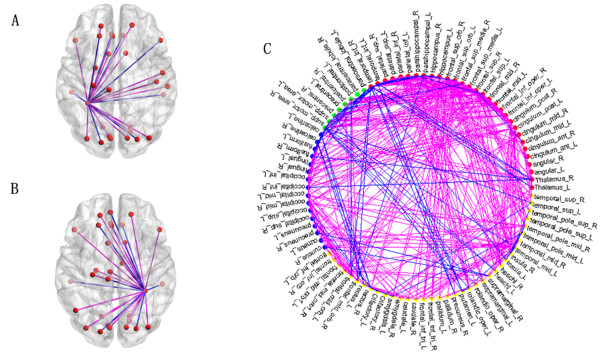
**The functional connections of the fusiform gyrus and the whole brain.** (**A**) is the functional connectivity between the left fusiform gyrus and other brain regions. (**B**) is the functional connectivity between the right fusiform gyrus and other brain regions. (**C**) is the 408 functional connections. The node represents the brain region. The line represents the related functional connection. The color of the node represents a specific network: blue represents the VN, red represents the DMN, green represents the SMN, and yellow represents the other regions. The color of the line signifies the alteration of the functional connections: blue denotes greater and purple denotes lesser.

As described above, on every fold of the LOOCV, we selected 550 of the highest-ranked features. Because feature ranking was based on a different subset of data for each iteration, the selected features differed with every LOOCV. However, 408 features were included in every fold of the LOOCV for the SVM classifier, resulting in 846 features that were represented across all folds of the LOOCV. We considered these 846 features to be the “participating” functional connections. The 408 functional connectivity’s map was graphed in Figure 4.

The 408 functional connectivity related to the brain regions primarily located within the visual cortical network (VN), an area that controls visual processing [24-26]; the DMN [27-35], which controls episodic memory[33] and self-projection [36]; the self-referential mental network(SRN), which plays an important role in self-referential mental activity [37]; and the sensory-motor network(SMN) [33,38] (Figure 4). In addition, there were some functional connections related to regions within the auditory network (AN), which regulates mood and affective processing [25,30,39].

### Brain regions with highly discriminative power

To evaluate the relative functional contribution of different brain regions to schizophrenia, we computed the value of the “region weight” for each brain region with the sum of their feature weights. We noted that there were several brain regions that exhibited greater weight than others. Furthermore, the fusiform gyrus exhibited the greatest weight. Alterations of functional connectivity of the fusiform gyrus were presented in Figure 4 and Table 2.

**Table 2:** Altered functional connectivity between fusiform gyrus and other brain regions

**Brain region A**	**Brain region B**	**Tau**
’fusiform_R’	'angular_L'	0.3017
	’calcarine_L'	0.5165
	'calcarine_R'	0.5413
	'cingulum_ant_L'	0.343
	'cingulum_mid_L'	0.442
	'cingulum_mid_R'	0.417
	’cuneus_L'	0.682
	'cuneus_R'	0.5868
	'frontal_inf_tri_L'	0.4504
	'frontal_inf_tri_R'	0.405
	'frontal_sup_L'	0.471
	'frontal_sup_media_L'	0.3388
	’frontal_sup_orb_L'	-0.4545
	'frontal_sup_orb_R'	-0.5909
	'hippocampus_L'	0.3017
	'lingual_L'	0.5372
	'lingual_R'	0.686
	'occipital_mid_L'	0.4793
	'occipital_mid_R'	0.5041
	'occipital_sup_L'	0.4835
	'occipital_sup_R'	0.5455
	'Olfactory_L'	-0.3058
	'Olfactory_R'	-0.5082
	'paracentral_lobule_L'	0.3884
	'parahippocampal_L'	-0.3512
	'rectus_L'	-0.5248
	'rectus_R'	-0.5661
	'temporal_inf_L'	-0.6488
	'temporal_inf_R'	-0.7066
’fusiform_L’	'amygdala_L'	0.3471
	'calcarine_L'	0.4711
	'calcarine_R'	0.376
	'cingulum_ant_L'	0.2975
	'cuneus_L'	0.7438
	'cuneus_R'	0.6446
	'frontal_inf_tri_L'	0.3306
	'frontal_inf_tri_R'	0.3223
	'frontal_sup_L'	0.3843
	'frontal_sup_orb_L'	-0.5702
	'frontal_sup_orb_R'	-0.6115
	'heschl_R'	0.3802
	'hippocampus_L'	0.4256
	'lingual_L'	0.4917
	'lingual_R'	0.5372
	'occipital_mid_L'	0.5165
	'occipital_mid_R'	0.5124
	'occipital_sup_L'	0.5703
	'occipital_sup_R'	0.5868
	'Olfactory_L'	-0.2934
	'Olfactory_R'	-0.3471
	'paracentral_lobule_L'	0.343
	'rectus_L'	-0.6735
	'rectus_R'	-0.686
	'rolandic_oper_R'	0.2893
	'temporal_inf_L'	-0.5909
	'temporal_inf_R'	-0.657
	'temporal_sup_R'	0.3967

## Discussion

This study demonstrated that schizophrenic patients might be discriminatively identified from healthy controls using whole-brain rs-fcMRI with an excellent generalization rate. In addition, we found that most of the identified brain regions located within the VN, the DMN and the SMN. Particularly, the bilateral fusiform gyrus exhibited the greatest weight, suggesting that aberrant functional connectivity of the fusiform gyrus with other brain regions might proportionally contribute to the impairment of the perception of emotions caused by facial stimuli in schizophrenia.

### Reliable identification of schizophrenia

**Figure 5:**
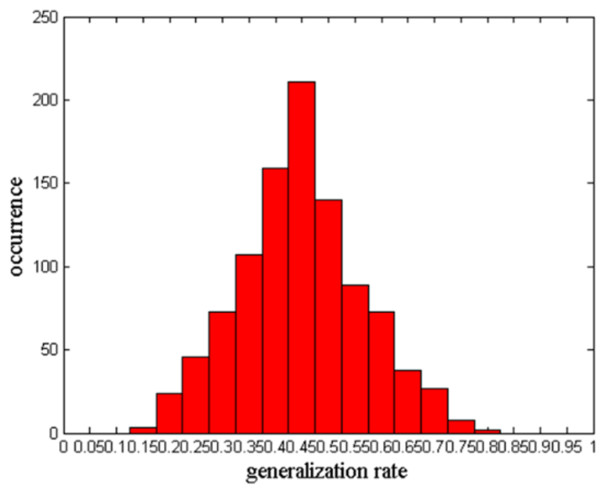
**Permutation distribution of the estimate using SVM (linear kernel).** The x-axis and y-axis labels are the GR and occurrence, respectively.

These years, some studies have attempted to identify biomarkers to differentiate schizophrenic patients from healthy controls [1,9,12,40-44]. To the best of our knowledge, the performance accuracy of the current classifier is approximately 85% [9,40,41]. Nevertheless, the overall generalization rate in this study achieved 93.2% (100% of healthy controls and 86.4% of schizophrenic patients, p < 0.001) by using 550 resting-state functional connectivity features. With generalization rate as the statistic, permutation distribution of the estimate was shown in Figure 5, which revealed that this classifier learned the relationship between the data and the labels with a risk of being wrong with probability of lower than 0.0001.

In this study, we used the linear method to reduce the dimension of the feature space and classify the subjects, and we found that the results were still satisfactory. Thus, whole-brain patterns based on rs-fcMRI may provide insight into our understanding of the pathophysiology of schizophrenia.

### Analysis of resting-state network

In this study, we observed that most of the functional connections characterized by a high discriminative power located within the VN(an area comprising regions including the precuneus, the fusiform gyrus, the lingual gyrus, the occipital cortex, and the calcarine gyrus), the DMN (including regions such as the inferior temporal gyrus, anterior cingulate cortex, middle cingulate gyrus, posterior cingulated cortex, superior parietal gyrus, hippocampus, parahippocampal gyrus, thalamus, and dorsomedial and medial prefrontal cortex) [27-32] and the SMN (including the precentral and postcentral gyri, the primary sensory-motor cortices, and the supplementary motor area). As shown in Figure 4, we found that, compared to the controls, some of the functional connectivity within or between these networks increased in patients diagnosed with schizophrenia and some of them decreased, which was consistent with the results obtained by several other studies [25,28,33,45]. For example, our results showed that the functional connectivity of ACC with the superior frontal cortex in DMN decreased in schizophrenic patients, which was in accordance with the result of the previous study [46]. In addition, we also found that the functional connectivity of superior temporal gyrus with the inferior frontal cortex in DMN increased in schizophrenic patients, which was consistent with the result of another study [47].

Overall, the abnormal functional connectivities within or between these networks might account for the cognitive and emotional disturbances, the abnormal filtering of visual information and dysfunction in working memory and motor and sensory control observed in schizophrenic patients [48-51].

### Analysis of the greater weight region

By reconstruction, we found that the fusiform gyrus, which played an important role in face, body, and word recognition, exhibited the greatest weight in this study. As shown in Table 2, the fusiform gyrus decreased the functional connections with the visual cortex areas including the calcarine, the lingual gyrus and the cuneus, indicating that the fusiform gyrus might be a core brain region in visual and cognitive process in schizophrenia[52,53]. We also found that fusiform gyrus decreased the functional connections with the hippocampus, parahippocampus and the amygdale, suggesting that the dysfunction of the fusiform gyrus might be related to the facial expressions and produce hallucinations of faces. Britton et al. [54] found the amygdale and fusiform had a within-run “U” response pattern of activity to facial expression blocks. Moreover, as shown in the Table 1, the logical memory scores of schizophrenia were lower than the controls. It suggested patients diagnosed with schizophrenia displayed logical deficits, which led to the symptoms such as hallucination and delusion. Several studies have demonstrated it [55,56], just as Hall et al. [57] found the patients with schizophrenia showed a relative lower overall connectivity between the fusiform gyrus and amygdale and Onitsuka et al. [58] found performance deficits on both immediate and delayed facial memory tests significantly correlated with the degree of bilateral anterior fusiform gyri activity reduction in patients with schizophrenia. Besides, we found the increased functional connection of fusiform gyrus with the orbital part of superior frontal cortex, the gyrus rectus and the inferior temporal gyrus in Table 2, just as Jafri et al. [53] and Zhou et al. [59] found that some functional connections increased in schizophrenia.

Accordingly, the dysfunctions of fusiform gyrus may be a very important core in visual and cognitive processing of facial and logical information in schizophrenia. Moreover, this result may provide insights into the identification of potentially effective biomarkers for the clinical diagnosis of schizophrenia.

### Reliability of the algorithm

#### LOOCV

Cross-validation is a commonly used technique for prediction and estimation of how accurately a predictive model will perform in practice. LOOCV can be a good choice when data available for test is not enough. LOOCV also works well for estimating generalization error. So, in the study, we used LOOCV to estimate generalized performance of our classifier. In LOOCV, the final feature set differed slightly from iteration to iteration. We finally analyzed the consensus functional connectivity which was defined as the functional connectivity feature appearing in every fold of the LOOCV. The changes in the consensus functional connectivity represent the common alternation in patients.

In addition, considering the restriction of our sample size which was relatively small, the three parameters such as number of features, dimension for PCA and C for SVM, were also estimated using LOOCV to achieve best performance of the classifier and greatest discrepancy between schizophrenia and control in resting-state functional connectivity. Naturally, if we had enough samples, we could get the optimized value of parameters in train data using LOOCV and predicted the test data using the optimized value of parameters. In paper, we reported best results which could obtain the difference functional connectivity between schizophrenia and control. However, as in Figure 3, even when the three parameters were not optimized, the worst result of classification we got was 75%, which was enough for discrimination of schizophrenia subjects from controls. Similar applications of LOOCV in obtaining the optimized parameters were seen in previous reports [18,20,60].

#### Permutation test

We also employed permutation test to assess the statistical significance of all the LOOCV results. For each LOOCV, the classification labels of the training data were randomly permuted 1000 times. With generalization rate as the statistic, the result of permutation distribution of the estimate revealed that this classifier learned the relationship between the data and the labels with a risk of being wrong with probability of lower than 0.0001. Similar evaluations of the algorithm performance were reported using permutation tests [20,21,23].

#### Limitations and future work

There are a few limitations in our current study. On the one hand, our sample size was relatively small. We wish to test our methods on a larger independent dataset to confirm our findings. On the other hand, participating schizophrenic patients were recruited from the inpatient hospital, so that patients were treated with antipsychotic medication during the data collection. Thus, it is possible that some of the differences found between schizophrenic patients and controls may relate to effects from medication. Furthermore, our data was collected in 1.5 T MRI, which may bring about the problem of the quality of image.

## Conclusion

In this study, we successfully differentiated schizophrenic patients from healthy controls and determined weighted brain regions by using whole-brain rs-fcMRI analysis. Our results demonstrate a good performance of the classification algorithm. Therefore, we believe that schizophrenic patients have dysfunctions in the visual cortical areas, the DMN, and the SMN, and the fusiform gyri play a very important role in the dysfunctions in patients diagnosed with schizophrenia.

## Abbreviations

CEN, Central-Executive Network; DMN, Default Mode Network; fMRI, functional Magnetic Resonance Imaging; BOLD, Blood Oxygen Level Dependent; LLE, Local Linear Embedding; PCA, Principle Component Analysis; SVM, Support Vector Machine; DSM-IV, Diagnostic and Statistical Manual of Mental Disorders, Fourth Edition; LOOCV, Leave-One-Out Cross-Validation; GR, Generalization Rate; SS, Sensitivity; SC, Specificity; VN, Visual Cortical Network; SRN, Self-Referential Mental Network; SMN, Sensory-Motor Network; AN, Auditory Network; rs-fcMRI, Resting-state functional connectivity Magnetic Resonance Imaging.

## Competing interests

The authors declare that we have no competing interests.

## Authors’ contributions

YT, LW and FC managed the literature searches and analyses and undertook the statistical analysis. YT and FC wrote the first draft of the manuscript. All authors read and approved the final manuscript.

## Supplementary Material

Additional file 1 The descriptions of PCA algorithm. Click here for file

Additional file 2 The descriptions of linear SVM algorithm. Click here for file
