# Supplementary material for: Identify schizophrenia using resting-state functional connectivity: an exploratory research and analysis
Source: Biomed Eng Online. 2012 Aug 16;11:50. doi: 10.1186/1475-925X-11-50 (PMC3462724; doi:10.1186/1475-925X-11-50)
Supplement: Additional file 1 — The descriptions of PCA algorithm. [file 1475-925X-11-50-S1.doc]

**Appendix A**

PCA is mathematically defined as an orthogonal linear transformation that transforms the data to a new coordinate system such that the greatest variance by any projection of the data comes to lie on the first coordinate, the second greatest variance on the second coordinate, and so on.

Define a data matrix, $X^{T}=(x_{1}$…$,x_{d})$, …$,\tilde{x}_{d})$, where d is the number of the reduced dimensionality. The PCA transformation that preserves dimensionality (that is, gives the same number of principal components as original variables) is then given by:

(A.1)

$U^{T}=(u_{1}$…$,u_{d})$was made up of $d$eigenvectors with the first $d$ largest eigenvalues of the covariance matrix. Since $U$ (by definition of the SVD of a real matrix) is an orthogonal matrix, each row of $\tilde{X}^{T}$ is simply a rotation of the corresponding row of $X^{T}$. The first column of $\tilde{X}^{T}$ is made up of the "scores" of the cases with respect to the "principal" component, the next column has the scores with respect to the "second principal" component, and so on. We chose 6 as the reduced dimension.

Reference:

1. http://en.wikipedia.org/wiki/Principal_component_analysis
