# Supplementary material for: Identify schizophrenia using resting-state functional connectivity: an exploratory research and analysis
Source: Biomed Eng Online. 2012 Aug 16;11:50. doi: 10.1186/1475-925X-11-50 (PMC3462724; doi:10.1186/1475-925X-11-50)
Supplement: Additional file 2 — The descriptions of linear SVM algorithm. [file 1475-925X-11-50-S2.doc]

**Appendix B**

Linear SVM

Given a set of training data

$D=\{(x_{i},y_{i})|x_{i}\in R^{p},y_{i}\in\{-1,1\}\}_{i-1}^{n}\}$, (B.1)

which include n points, Where the $y_{i}$ is either 1 or -1, indicating the class to which the point $x_{i}$ belongs. Each $x_{i}$ is a p-dimensional real vector. We want to find the maximum-margin hyperplane that divides the points having $y_{i}=1$from those having$y_{i}=-1$.These hyperplanes can be described by the equations

$w\cdot x-b=1$ (B.2)

And

$w\cdot x-b=-1$ (B.3)

By using geometry, the distance between these two hyperplanes is$\frac{2}{\left\|w\right\|}$. The goal of a linear SVM classifier is to find a decision function$f(x)=w'x+b$ by solving the following optimization problem:

$$\min_{w_{i}\xi}$$

$$\frac{1}{2}\left\|w\right\|^{2}+C\sum_{i=1}^{N} \xi_{i}$$

$s.t.$ $y_{i}(w'x_{i}+b)\geq1-\xi_{i}$ (B.4)

where $w$ is the normal of the hyperplane; $\xi_{i}$are a measure of the misclassification errors for non-separable cases; and C trades off the empirical risk and model complexity (we set C=0.255 for all cases).

Reference:

1. http://en.wikipedia.org/wiki/Support_vector_machine
